# PolyQ Repeat Expansions in *ATXN2* Associated with ALS Are CAA Interrupted Repeats

**DOI:** 10.1371/journal.pone.0017951

**Published:** 2011-03-29

**Authors:** Zhenming Yu, Yongqing Zhu, Alice S. Chen-Plotkin, Dana Clay-Falcone, Leo McCluskey, Lauren Elman, Robert G. Kalb, John Q. Trojanowski, Virginia M.-Y. Lee, Vivianna M. Van Deerlin, Aaron D. Gitler, Nancy M. Bonini

**Affiliations:** 1 Department of Biology, University of Pennsylvania, Philadelphia, Pennsylvania, United States of America; 2 Howard Hughes Medical Institute, Philadelphia, Pennsylvania, United States of America; 3 Department of Cell and Developmental Biology, University of Pennsylvania School of Medicine, Philadelphia, Pennsylvania, United States of America; 4 Department of Neurology, University of Pennsylvania School of Medicine, Philadelphia, Pennsylvania, United States of America; 5 Center for Neurodegenerative Disease Research, University of Pennsylvania School of Medicine, Philadelphia, Pennsylvania, United States of America; 6 Department of Pathology and Laboratory Medicine, University of Pennsylvania School of Medicine, Philadelphia, Pennsylvania, United States of America; 7 Department of Pediatrics, The Children's Hospital of Philadelphia, Philadelphia, Pennsylvania, United States of America; University Medical Center Groningen, University of Groningen, The Netherlands

## Abstract

Amyotrophic lateral sclerosis (ALS) is a devastating, rapidly progressive disease leading to paralysis and death. Recently, intermediate length polyglutamine (polyQ) repeats of 27–33 in *ATAXIN-2* (*ATXN2*), encoding the ATXN2 protein, were found to increase risk for ALS. In *ATXN2*, polyQ expansions of ≥34, which are pure CAG repeat expansions, cause spinocerebellar ataxia type 2. However, similar length expansions that are interrupted with other codons, can present atypically with parkinsonism, suggesting that configuration of the repeat sequence plays an important role in disease manifestation in *ATXN2* polyQ expansion diseases. Here we determined whether the expansions in *ATXN2* associated with ALS were pure or interrupted CAG repeats, and defined single nucleotide polymorphisms (SNPs) rs695871 and rs695872 in exon 1 of the gene, to assess haplotype association. We found that the expanded repeat alleles of 40 ALS patients and 9 long-repeat length controls were all interrupted, bearing 1–3 CAA codons within the CAG repeat. 21/21 expanded ALS chromosomes with 3CAA interruptions arose from one haplotype (GT), while 18/19 expanded ALS chromosomes with <3CAA interruptions arose from a different haplotype (CC). Moreover, age of disease onset was significantly earlier in patients bearing 3 interruptions vs fewer, and was distinct between haplotypes. These results indicate that CAG repeat expansions in *ATXN2* associated with ALS are uniformly interrupted repeats and that the nature of the repeat sequence and haplotype, as well as length of polyQ repeat, may play a role in the neurological effect conferred by expansions in *ATXN2*.

## Introduction

Amyotrophic lateral sclerosis (ALS, also referred to as Lou Gehrig's disease) is a progressive, fatal neurodegenerative disease caused by the degeneration of motor neurons [1]–[3]. Approximately 10% of ALS cases are familial, with the remainder of cases being sporadic. To date, 12 genetic loci have been identified associated with familial ALS. Among these, mutations in superoxide dismutase 1 (*SOD1*) account for 15–20% of cases of familial ALS [4]. Trans- activate reponse DNA-binding protein (TARDBP, or TDP-43) is a major disease protein of the ubiquitin-positive cytoplasmic inclusions in ALS without SOD1 mutations [5]. Mutations in the TDP-43 coding gene *TARDBP* were later found in multiple cases of familial and sporadic ALS [6]–[9], indicating that TDP-43 plays a critical role in disease pathogenesis.

Spinocerebellar ataxia 2 (SCA2) is an autosomal dominant disease caused by an expanded CAG trinucleotide repeat encoding glutamine within the open reading frame of the gene encoding the ataxin 2 protein, *ATAXIN2* (*ATXN2*) [10]–[12]. As with other polyglutamine (polyQ) diseases, the length of the CAG repeat expansion is inversely correlated with disease onset and severity [13]. CAG repeats in normal alleles of *ATXN2* are variable in length, although by far the most common allele carries 22 repeats. In the normal *ATXN2* allele, the CAG repeat region encoding the glutamine domain is typically interrupted by one or more CAA repeats (also encoding glutamine). The CAG repeat region within expanded alleles of *ATXN2* associated with SCA2 disease are 34–59 in length [10]–[12], [14]–[19]. Similar to other polyQ diseases, SCA2 is for the most part a “pure” repeat disease. That is, the expanded allele is comprised of an uninterrupted sequence of CAGs encoding glutamine. However, four years after the 1996 identification of expansions in *ATXN2* as the molecular basis of SCA2, a pathogenic length expansion bearing an “interrupted” repeat sequence was first reported [20]. It is now recognized that lower-range polyQ repeat expansions in *ATXN2*, of 33–49 in length, can be associated with levo-dopa responsive parkinsonism [21]–[30]. Intriguingly, in the situations of parkinsonism sequenced to date, the CAG repeat region has been found to not be a pure CAG repeat run, but rather to be interrupted with one or more CAA (or other) codons [21]–[30]. Additional data suggests that two SNPs (rs695871 and rs695872) in exon 1 of the *ATXN2* gene, where the CAG repeat region occurs, predominant in distinct patterns in normal versus SCA2 individuals: the GT haplotype predominates in controls, whereas the CC haplotype is associated with disease [19].

Recently, intermediate-length polyQ repeats in *ATXN2* were found to be a significant risk factor for ALS [31]. Consistent with the association between *ATXN2* and ALS, it was previously known that SCA2 can present with motor neuron features that mimic ALS [32], [33]. Thus, it appears that individuals with repeat expansions in *ATXN2* can present with SCA2, parkinsonism, or ALS depending upon the length of the repeat. Moreover, the finding that *ATXN2*-linked parkinsonism may be associated with an interrupted CAG repeat region, versus the pure CAG repeats typical of SCA2, raises the possibility that the CAG purity of the repeat expansion may contribute to disease manifestation.

Given these data, we sequenced the polyQ repeat region within *ATXN2* in the cases of ALS and controls harboring ATXN2 polyQ repeats of 27 and higher [31]. Our results show that the repeats associated with increased risk for ALS are uniformly comprised of interrupted CAG repeats. All of the sequences are interrupted by 1–3 CAA codons; none of the repeats harbors a pure CAG repeat. Further, SNP analysis using the derived cleaved amplified polymorphic sequences (dCAPS) technique [34] revealed that the alleles with 3 CAA codons are the GT haplotype, whereas alleles with fewer CAA codons are predominantly the CC haplotype. Age of disease onset in subjects with ALS and 3 CAA repeat interruptions, compared to those with fewer, revealed that the age of onset was significantly earlier; age of disease onset also differed by haplotype. This is despite the fact that the average repeat length is shorter in subjects with 3 CAA interruptions. These results highlight that an interplay of the CAG repeat sequence configuration, haplotype association, as well as repeat length, may play a prominent role in disease manifestation in *ATXN2*-associated neuropathologies.

## Results

### CAA interruptions in the CAG repeat region of *ATXN2* in ALS

We characterized the repeat sequence in 40 of the 45 ALS cases with intermediate length polyQ repeats in the *ATXN2* gene of 27–33 in length and nine of the control cases [31]; these constituted a subset of 40 ALS cases we previously reported with expanded *ATXN2* alleles for which motor neuron disease was the initial presentation and from which we were able to obtain amplifiable DNA. All 40 cases met El Escorial criteria for ALS; in addition, for 19/40 cases (those from the University of Pennsylvania Center for Neurodegenerative Disease Research (CNDR)) clinical charts were available and reviewed by a neurologist to confirm the diagnosis of ALS. With the exception of one previously described individual with ataxic features late in the course of disease (case A14, Table 1; [31]), these 19 well-characterized cases did not demonstrate features atypical of ALS such as parkinsonism or ataxia.

**Table 1:** Sequence of the polyQ repeat region of expanded allele of ALS patients.

Case ID	Expanded allele	Age at Onset	CAG Pattern	SNP expanded allele**	CAA#	Pattern
A32	29	49	20-8	CC	1	OOOOOOOOOOOOOOOOOOOO•OOOOOOOO
A48	29	72	20-8	CC	1	OOOOOOOOOOOOOOOOOOOO•OOOOOOOO
A35	30	62	21-8	CC	1	OOOOOOOOOOOOOOOOOOOOO•OOOOOOOO
A33	30	72	21-8	CC	1	OOOOOOOOOOOOOOOOOOOOO•OOOOOOOO
A42	31	74	21-9	CC	1	OOOOOOOOOOOOOOOOOOOOO•OOOOOOOOO
A37	31	61	21-9	CC	1	OOOOOOOOOOOOOOOOOOOOO•OOOOOOOOO
A43	31	58	22-8	CC	1	OOOOOOOOOOOOOOOOOOOOOO•OOOOOOOO
A50	32	77	22-9	CC	1	OOOOOOOOOOOOOOOOOOOOOO•OOOOOOOOO
A47	32	79	23-8	CC	1	OOOOOOOOOOOOOOOOOOOOOOO•OOOOOOOO
A51	32	55	23-8	CC	1	OOOOOOOOOOOOOOOOOOOOOOO•OOOOOOOO
A49	32	53	23-8	CC	1	OOOOOOOOOOOOOOOOOOOOOOO•OOOOOOOO
A53	33	52	23-9	CC	1	OOOOOOOOOOOOOOOOOOOOOOO•OOOOOOOOO
A52	33	70	23-9	CC	1	OOOOOOOOOOOOOOOOOOOOOOO•OOOOOOOOO
A30	28	55	13-5-8	CC	2	OOOOOOOOOOOOO•OOOOO•OOOOOOOO
A41 *	29	35	13-6-8	CC	2	OOOOOOOOOOOOO•OOOOOO•OOOOOOOO
A34	30	58	13-7-8	CC	2	OOOOOOOOOOOOO•OOOOOOO•OOOOOOOO
A38	31	56	8-13-8	GT	2	OOOOOOOO•OOOOOOOOOOOOO•OOOOOOOO
A40	31	65	13-7-9	CC	2	OOOOOOOOOOOOO•OOOOOOO•OOOOOOOOO
A45	32	54	13-8-9	CC	2	OOOOOOOOOOOOO•OOOOOOOO•OOOOOOOOO
A14	27	30	8-4-4-8	GT	3	OOOOOOOO•OOOO•OOOO•OOOOOOOO
A17 *	27	65	8-4-4-8	GT	3	OOOOOOOO•OOOO•OOOO•OOOOOOOO
A16	27	not known	8-4-4-8	GT	3	OOOOOOOO•OOOO•OOOO•OOOOOOOO
A18	27	53	8-4-4-8	GT	3	OOOOOOOO•OOOO•OOOO•OOOOOOOO
A19	27	59	8-4-4-8	GT	3	OOOOOOOO•OOOO•OOOO•OOOOOOOO
A20	27	57	8-4-4-8	GT	3	OOOOOOOO•OOOO•OOOO•OOOOOOOO
A21	27	60	8-4-4-8	GT	3	OOOOOOOO•OOOO•OOOO•OOOOOOOO
A22	27	46	8-4-4-8	GT	3	OOOOOOOO•OOOO•OOOO•OOOOOOOO
A23	27	49	8-4-4-8	GT	3	OOOOOOOO•OOOO•OOOO•OOOOOOOO
A24	27	53	8-4-4-8	GT	3	OOOOOOOO•OOOO•OOOO•OOOOOOOO
A12	27	66	8-4-4-8	GT	3	OOOOOOOO•OOOO•OOOO•OOOOOOOO
A25	27	48	8-4-4-8	GT	3	OOOOOOOO•OOOO•OOOO•OOOOOOOO
A27	27	51	8-4-4-8	GT	3	OOOOOOOO•OOOO•OOOO•OOOOOOOO
A26	27	47	8-4-4-8	GT	3	OOOOOOOO•OOOO•OOOO•OOOOOOOO
A28	27	62	8-4-4-8	GT	3	OOOOOOOO•OOOO•OOOO•OOOOOOOO
A10	27	51	8-4-4-8	GT	3	OOOOOOOO•OOOO•OOOO•OOOOOOOO
A11	27	61	8-4-4-8	GT	3	OOOOOOOO•OOOO•OOOO•OOOOOOOO
A13	27	59	8-4-4-8	GT	3	OOOOOOOO•OOOO•OOOO•OOOOOOOO
A31	29	64	8-4-4-10	GT	3	OOOOOOOO•OOOO•OOOO•OOOOOOOOOO
A39	31	63	8-4-8-8	GT	3	OOOOOOOO•OOOO•OOOOOOOO•OOOOOOOO
A46	32	42	8-4-9-8	GT	3	OOOOOOOO•OOOO•OOOOOOOOO•OOOOOOOO

*familial situation,

**SNP695871 SNP695872.

The *ATXN2* repeat region was amplified by polymerase chain reaction from genomic DNA samples. Because the repeats were intermediate in length compared to the normal allele (22 or 23), it was difficult to cleanly separate the allele bearing the longer repeat from the normal allele. Thus, we were unable to sequence the DNA directly from the PCR product and determine the sequence with high integrity. Instead, the amplification products were separated by size on a 4% agarose gel, then extracted, and expanded alleles subcloned and sequenced. Multiple clones for each allele were sequenced to assure against PCR-introduced changes.

The length of the normal allele of the ALS patients was typically 22; this is consistent with previous findings that a repeat of 22 is the most common normal allele [16], [17], [19], [35]. For the longer alleles, in ALS patients (n = 40 with *ATXN2* expansion), the repeats ranged from 27 to 33, and in controls (n = 9 with *ATXN2* expansions) from 27 to 32. Sequencing of the repeat region revealed that none of the polyQ repeats in patients with ALS were comprised of pure CAG repeat expansions. Rather, all were interrupted by at least 1 CAA codon, in stark contrast to the situation observed in SCA2. Interruptions of the repeat sequence are typical of the intermediate expansion repeats observed in control individuals [19]. Note that such interruptions do not affect the protein sequence, as CAA also encodes glutamine. Analysis of the repeat length patterns indicated that the repeat region was interrupted by 1, 2 or 3 CAA codons. Aside from the striking finding that none of the repeats were pure CAG repeats, we also observed among the Q27 repeats a monomorphic sequence pattern of 3 CAA interruptions in an 8-4-4-8 configuration.

In order to determine whether or not the polyQ repeat sequence was distinct among patients with repeat lengths within the ALS-susceptibility range and controls, we also sequenced 9 neurologically normal individuals with repeat lengths of 27 and longer (Table 2). This analysis confirmed that the polyQ-encoding DNA sequence was also interrupted in controls, and, moreover, bore interruption patterns found among the ALS patients. Thus, the controls of 27 had the same repeat configuration as the ALS patients; there was also no distinguishing feature among the controls with repeat lengths of 29, 30 and 32—these DNAs showed interrupted repeat patterns also found among ALS patients. Taken together, these data indicate that intermediate-length polyQ repeats in the *ATXN2* gene associated with ALS are CAA interrupted repeats bearing interruptions of 1, 2 or 3 CAA codons.

**Table 2:** Sequence of the polyQ repeat region of expanded alleles of controls.

Case ID	Atx2 length	Age at sampling	CAG Pattern	SNP expanded allele	CAA #	Pattern
N11	27	80	8-4-4-8	GT	3	OOOOOOOO•OOOO•OOOO•OOOOOOOO
N12	27	65	8-4-4-8	GT	3	OOOOOOOO•OOOO•OOOO•OOOOOOOO
N13	27	62<1/emph>	8-4-4-8	GT	3	OOOOOOOO•OOOO•OOOO•OOOOOOOO
N14	27	61	8-4-4-8	GT	3	OOOOOOOO•OOOO•OOOO•OOOOOOOO
N15	27	74	8-4-4-8	GT	3	OOOOOOOO•OOOO•OOOO•OOOOOOOO
N16	27	73	8-4-4-8	GT	3	OOOOOOOO•OOOO•OOOO•OOOOOOOO
N22	29	59	8-4-4-10	GT	3	OOOOOOOO•OOOO•OOOO•OOOOOOOOOO
N23	30	57	21-8	CC	1	OOOOOOOOOOOOOOOOOOOOO•OOOOOOOO
N24	32	59	13-8-9	CC	2	OOOOOOOOOOOOO•OOOOOOOO•OOOOOOOOO

**SNP695871 SNP695872.

### Association of the haplotype of rs695871 and rs695872 with CAA interruptions in *ATXN2*

**Figure 1:**
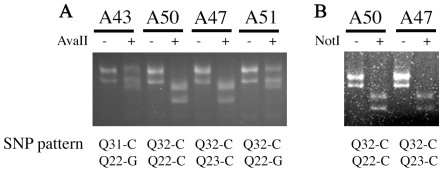
SNP analysis by dCAPS method. Analysis of restriction fragments for SNP (A) rs695871 and (B) rs695872. For SNP rs695871 (C or G, AvaII digestion) analysis of a Q32/Q22 patient, the amplified products were Q32:359bp and Q22:329bp. If Q32 is linked to a C, AvaII will digest the product into two fragments of 313 bp and 46 bp. If Q32 is linked to a G, AvaII will not cut. If Q22 is linked to a C, AvaII will digest it into fragments of 46 bp and 283 bp. If Q22 is linked to a G, AvaII will not cut. In the case of C/G heterozygotes, some uncut hybrid product remained. For SNP rs695872 (C or T, NotI digestion) analysis of a Q32/Q22 patient, the amplified products were Q32:308bp and Q22:278bp. If Q32 is linked to a C, NotI will digest the product into two fragments of 254 bp and 64 bp. If Q32 is linked to a T, NotI will not cut. If Q22 is linked to a C, NotI will digest it into fragments of 64 bp and 214 bp. If Q22 is linked to a T, NotI will not cut. In the case of C/T heterozygotes, some uncut hybrid product remained. In the gels, the low molecular weight bands (46, 64 bp) are largely undetectable; the pattern is scored by whether the higher bands are cut or not.

Two SNPs are found in exon 1 which bears the polyQ domain of *ATXN2*. rs695871 is 177 bp upstream of the repeat, and bears either a G or C base; the polymorphism affects the amino acid sequence, changing a Val to a Leu. The second SNP rs695872 is a T or C, and is silent, with no effect on the protein sequence. Others have previously reported that these two SNPs define two distinct haplotypes, a CC haplotype that appears to be evolutionarily ancestral and a GT haplotype of more recent origin; the CC haplotype predominates among SCA2 expanded alleles [19], [36]. Using dCAPS, to introduce a restriction enzyme site depending upon the polymophism to allow the SNPs to be defined by polymerase chain amplification followed by restriction enzyme analysis [34], we defined these polymorphisms in the ALS patients and control cases (Figure 1; Tables 1 and 2). This analysis showed that the 1 and 2 CAA interruption situations were predominantly associated with the CC haplotype (18/19 expanded chromosomes), whereas the 3 CAA interrupted alleles were uniformly the GT haplotype (21/21 chromosomes). This distinction was also the case for the intermediate length alleles among the controls.

### Association of the number of CAA interruptions and haplotype in *ATXN2* with age of disease onset among the ALS patients

**Figure 2:**
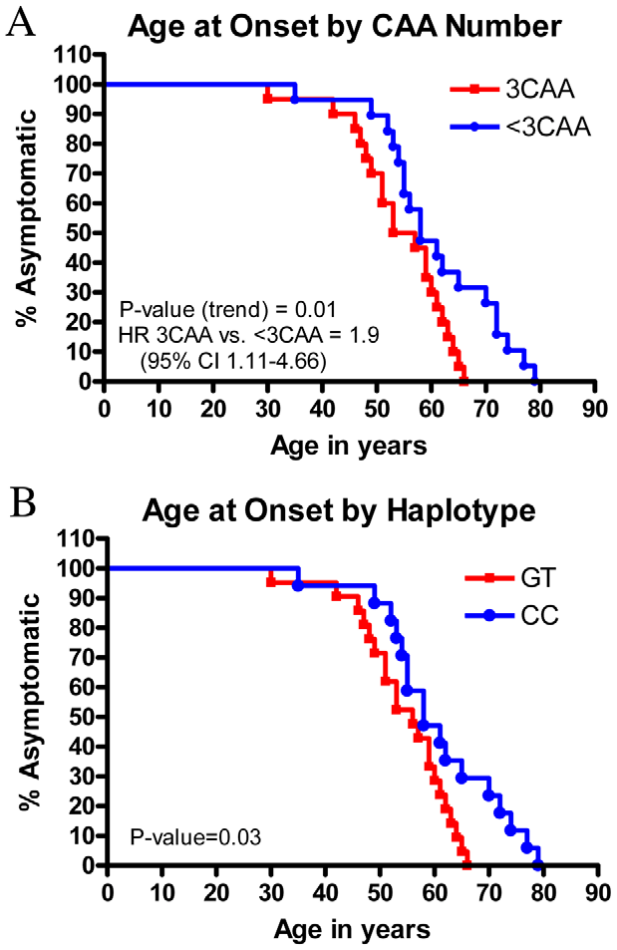
Age of disease onset by repeat interruptions and haplotype. Age of disease onset by (A) repeat interruptions or by (B) haplotype for ALS patients bearing either 3CAA interruptions within the polyQ repeat region, versus patients with fewer than 3 CAA interruptions. The patients with 3 interruptions show an earlier disease onset, with a hazard ratio (HR) of 1.9, compared to patients with fewer interruptions. This is despite that patients with 3 CAA interruptions have an average repeat length that is lower (27.5) compared to patients with fewer interruptions (30.8). The age of disease onset is also significantly different when broken down by haplotype.

We considered whether the number of CAA interruptions within the repeat region and/or the genetic background in the form of SNP haplotype might result in differences in disease features among the 40 ALS patients. We evaluated whether age at onset, disease duration (for postmortem cases, n = 15), gender and presence/absence of atypical features differed by number of CAA interruptions or SNP haplotype. We found that age at onset among the ALS patients differed significantly depending on the number of CAA interruptions and the haplotype in the expanded allele, with individuals with more CAA interruptions and of the GT haplotype developing disease earlier (Figure 2, number of interruptions p-value for trend = 0.01, 3CAA vs <3CAA HR 1.9; haplotype, p = 0.03; log rank test). This is despite the fact that ALS patients with 3CAA interruptions have a shorter average repeat length (27.5) versus patients with fewer interruptions (30.8). Normally, for polyQ repeat expansion diseases, the relationship between repeat length and disease onset is such that longer repeats are associated with an earlier age of disease onset [13], [37]; thus, this trend for an earlier age of disease onset does not correlate with average repeat length. Other clinical features, such as gender, disease duration, presence/absence of atypical features, did not differ among the groups.

## Discussion

### Multiple faces of *ATXN2* in neurological disease

**Figure 3:**
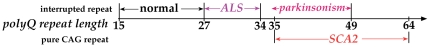
The spectrum of diseases associated with ATXN2 polyQ repeat expansions. Although the repeat in *ATXN2* is polymorphic normally, it is typically 22. In this cohort of controls, we found that only 1.4% (12/980 total) had repeats greater than 27 (range 27–31), whereas 4.7% of this cohort of ALS patients [31] had repeats of 27–33 (43/915 sporadic, 3/65 familial); the exact cut-off associated with risk for ALS may vary in different populations [38]. The repeats of ALS and high range controls from this study are interrupted by 1–3 CAA codons. *ATXN2* repeats associated with parkinsonism range from 34 to 49, and studies to date indicate that the repeats are interrupted [21], [27]. Repeat expansions associated with SCA2 are greater than 34, and are typically pure CAG repeats [12], [14], [37].

PolyQ expansions in ATXN2 are now associated with three clinically distinct neurological diseases; these different situations are associated with different repeat lengths and sequence configuration of the DNA repeat region (Figure 3). If the polyQ domain within *ATXN2* is composed of a pure CAG repeat with a length ≥34, this presents with SCA2 [10]–[12], [14]–[19]. However, repeat expansions in *ATXN2* can also present with levo-dopa responsive parkinsonism; about half of these situations have been sequenced. The repeat lengths range from 33 to 49, and in the situations sequenced, the polyQ domain is not a pure CAG repeat run, but rather is comprised of an interrupted CAG repeat region [22]–[30]. Further, recent studies indicate that intermediate-length polyQ repeats in *ATXN2* of 27 to 33 are associated with increased risk for ALS [31], although the precise repeat length cut-off is likely to vary depending upon the population [38]. Here we show that ALS-associated polyQ repeats in *ATXN2* are uniformly interrupted CAG repeats, comprised of interruptions of 1–3 CAA codons. We also observed a haplotype distinction between individuals bearing 3CAA interruptions and fewer. Note that CAA also encodes glutamine, thus the repeat region within the protein will remain a pure polyQ domain. These data suggest that the length of the polyQ repeat, as well as the CAG-repeat purity of the repeat region and the haplotype, influence disease presentation.

### RNA toxicity in *ATXN2*-related neuropathology

That interrupted and uninterrupted polyQ repeat expansions with similar length in *ATXN2* lead to different neurological presentations (SCA2 or parkinsonism) highlights the possibility that repeat sequence configuration, as well as repeat length, is an important arbiter in clinical manifestation and phenotypic variability in *ATXN2*-associated pathologies. SCA2 is known to show nigral atrophy and motor neuron loss; that is, the clinical manifestations of parkinsonism and motor neuron disease are within the spectrum of SCA2 pathology [39]–[41]. Intriguingly, select situations appear biased strongly toward one neurological outcome or another; interestingly, this may be influenced by not only the length of the repeat, but also the purity of the CAG repeat region encoding the polyQ expansion.

PolyQ disease is classically viewed as a purely protein-based disease, with dominant toxicity conferred by the expanded polyQ domain [13], [37]. However, some data suggest that the situation may be more complicated, and that pathogenic mechanisms beyond toxicity or abnormal interactions solely due to the polyQ-containing protein may contribute to polyQ expansion diseases. For example, toxicity at the level of the expanded repeat RNA is conferred by CAG expansions in *Drosophila* [42], *C elegans* and mammals [43], [44]. Notably, CAA interruptions within the repeat sequence for SCA3 shift the toxicity curve, such that expression of a polyQ protein of identical amino acid sequence, but encoded by a CAA/G interrupted repeat region, is less toxic [42]. In the context of human disease, interruptions may present with different manifestations, or different clinical features of disease being dominant. In potential support of this idea, interruption of the CAG repeat expansion by a CAT codon within the *ATXN1* polyQ-encoding domain was reported to cause later onset and milder disease in a rare case of SCA1 compared to pure, uninterrupted repeats of similar length [45]. An intriguing situation also occurs in SCA17, which is due to polyQ repeat expansions in TATA-box binding protein (TBP) [46]–[48]. Normally, the polyQ repeat region within TBP is interrupted by multiple CAA codons, and remains so in the expanded disease situation. Intriguingly, the threshold for a pathogenic repeat length is higher for repeat expansions for SCA17 (∼44) compared to that of most of the dominantly inherited spinocerebellar ataxias (∼37) [13], [49]. It is tempting to speculate that this shifted toxicity curve for SCA17 may be due to the interrupted repeat sequence of the polyQ domain within *TBP*.

Our findings are consistent with the idea that both the presence of any CAA interruptions and the presence of increasing numbers of CAA interruptions may influence phenotypic presentation. Specifically, while the overwhelming majority of SCA patients have pure CAG repeat expansions [10]–[12], [37], [41], 40/40 ALS patients in this study had at least one CAA interruption. Among these 40 patients with CAA interruptions, the number of interruptions as well as the haplotype may matter. Here we found an earlier age at onset for those ALS patients with more interruptions and who also share the GT haplotype.

The 3CAA-interrupted chromosomes arose on one genetic background (the GT haplotype), and most <3CAA-interrupted chromosomes arose on a different genetic background (the CC haplotype). We found both an association between earlier age at onset and number of CAA interruptions as well as SNP haplotype. Moreover, the fact that the ALS patients are not associated with a single haplotype may explain why ATXN2 has not emerged as a significant contributor to ALS from genome wide association (GWA) studies [50]–[53]. Intriguingly, however, a SNP linked to a gene that interacts with ATXN2, Ataxin-2 binding protein, has been identified as significant [50], [54]. Our previous study found an earlier age of disease onset in a small cohort of ALS patients (n = 65), when comparing those bearing a longer ATXN2 repeat to those with a normal length repeat [31]; it is possible that those data were driven by patients with 3CAA interruptions in the repeat sequence, of the GT haplotype. As with all such findings, such trends require confirmation among larger and additional sets of patients.

How might the sequence within the repeat region influence disease presentation? Whether the repeat sequence is a pure CAG or an interrupted CAG is predicted to influence somatic instability of the repeat, with a pure repeat being far more unstable and tending to expand [19], [55]–[57]. CAA interruptions have been proposed to play a critical role in conferring stability to the CAG repeat in *ATXN2*, with their absence predisposing *ATXN2* alleles towards instability and pathogenic expansion to SCA2 disease [19]. Interruptions in a CAG repeat RNA by CAA codons are predicted to affect the secondary structure [58]. Even the interruption of a single CAA codon can have a notable effect on structure and free energy of folding. Moreover, if, as with other expanded repeat RNAs such as CGG expanded RNA of FXTAS [59], [60] and expanded CTG repeats of myotonic dystrophy [61], the CAG repeat RNA interacts with select proteins, the altered structure due to CAA interruptions may result in differential interactions. Such differential protein or other interactions may in part contribute to the distinct disease manifestations. The purity of the repeat may also influence the level of the mRNA and/or level of the translated protein. Such an influence of the RNA may be notable for ALS, as defects in RNA processing and metabolism may particularly relevant. The association of mutations in TDP43 and fused in sarcoma (FUS)/translocated in liposarcoma (TLS), two RNA-binding proteins with striking structural and functional similarities [62], as well as ATXN2, which itself is an RNA binding protein, in ALS [31], suggests a prominent role for RNA binding proteins and RNA-dependent processes in the disease.

## Methods

### Ethics statement

Samples from the CNDR were obtained with written informed consent under Institutional Board Approval of the University of Pennsylvania. Samples from Coriell were obtained with permission from the Coriell Institute for Medical Research Institutional Review Board. The individual submitters who contributed DNA samples to Coriell received written informed consent from all patients (or guardians of patients) participating in the study (consent for research).

### PolyQ repeat amplification and sequencing

The polyglutamine repeat region of the *ATXN2* gene was amplified from genomic DNA isolated from B-lymphocytes of controls and ALS patients. Patient samples were from the CNDR (19/40 ALS samples with 27 or more repeats) and Coriell (21/40 ALS samples with 27 or more repeats), controls were from Coriell; for additional details see [31]. Amplification was carried out with the primers SCA2-S2 (5′-CGCCGCGTTCCGGCGTCTCC-3′) and SCA2-B (5′-CGGGCTTGCGGACATTGG-3′), using Takara LA taq with GC buffer (Takara RR02AG, Shiga, Japan) plus 5% DMSO (Sigma, Cat# D8779). The amplification products were separated on a 4% agarose gel, purified, then sub-cloned into the pGEMT Easy vector (Promega Cat.# A1360, Madison, Wisconsin) and transformed into DH5alpha competent cells (Invitrogen Cat. #18265017, Carlsbad, CA). Colonies were screened by amplification to identify those bearing the expanded allele. For each expanded allele, 2–6 clones from at least two independent PCR reactions were prepared by Qiagen mini-prep and sequenced using the T7 primer from the pGEMT Easy vector.

### SNP genotyping

The dCAPS technique [34] was used to analyze SNP rs695871 and SNP rs695872 (www.ncbi.nlm.nih.gov/SNP/) within exon1 of the human *ATXN2* gene. For SNP695871, Primer SCA2-AVAII (5′-ctcccggcggctccttggtctcggcggg CCTCCCCGCCCCTTCGT**G**GTC-3′, mismatch underlined) was used such that one mismatch was introduced to generate a recognition site for the restriction endonuclease AvaII (recognition site 5′-G′G(A/T)CC-3′). Products covering the SNP and polyQ repeat region were amplified with primers SCA2-AVAII and SCA2-B (5′-CGGGCTTGCGGACATTGG-3′) using LA taq DNA polymerase as above. Undigested and AvaII-digested products were separated on a 4% agarose gel in parallel. AvaII could only cut the amplified product if the SNP were a C and not a G. Thus, the SNP (C or G) and linkage to the normal or expanded allele were determined by the digestion pattern. For SNP695872, Primer SCA2-NOTI (5′-ccttctccccctcgccagcccgggcgcccctccggccgcgccaacccgcg CCTCCCCGCTCGGCG**G**CCG-3′, mismatch underlined) was used such that one mismatch was introduced to generate a recognition site for the restriction endonuclease NotI (recognition site 5′-GC′GGCCGC-3′). Products spanning the SNP and polyQ region were amplified with primers SCA2-NOTI and SCA2-B. Undigested and NotI-digested products were separated on a 4% agarose gel in parallel. NotI could only cut the amplified product if the SNP were a C and not T. Thus, the SNP (C or T) and the linkage to the normal or expanded allele were determined by the digestion pattern.

### Statistics

Survival curve analyses using log-rank tests were used to compare age at onset, disease duration, gender, and presence/absence of atypical features between subgroups of ALS patients with 1,2 and 3CAA interruptions, and between subgroups of ALS patients with different genetic backgrounds.
